# *In-silico* Taxonomic Classification of 373 Genomes Reveals Species Misidentification and New Genospecies within the Genus *Pseudomonas*

**DOI:** 10.3389/fmicb.2017.01296

**Published:** 2017-07-12

**Authors:** Phuong N. Tran, Michael A. Savka, Han Ming Gan

**Affiliations:** ^1^Genomics Facility, Tropical and Medicine Biology Platform, Monash University Malaysia Bandar Sunway, Malaysia; ^2^School of Science, Monash University Malaysia Bandar Sunway, Malaysia; ^3^Thomas H. Gosnell School of Life Sciences, Rochester Institute of Technology Rochester, NY, United States; ^4^Centre for Integrative Ecology, School of Life and Environmental Sciences, Deakin University Geelong, VIC, Australia

**Keywords:** *Pseudomonas*, taxonomy, phylogenomics, *in-silico* genome-genome hybridization, comparative genomics, average nucleotide identity, genospecies

## Abstract

The genus *Pseudomonas* has one of the largest diversity of species within the Bacteria kingdom. To date, its taxonomy is still being revised and updated. Due to the non-standardized procedure and ambiguous thresholds at species level, largely based on 16S rRNA gene or conventional biochemical assay, species identification of publicly available *Pseudomonas* genomes remains questionable. In this study, we performed a large-scale analysis of all *Pseudomonas* genomes with species designation (excluding the well-defined *P. aeruginosa*) and re-evaluated their taxonomic assignment via *in silico* genome-genome hybridization and/or genetic comparison with valid type species. Three-hundred and seventy-three pseudomonad genomes were analyzed and subsequently clustered into 145 distinct genospecies. We detected 207 erroneous labels and corrected 43 to the proper species based on Average Nucleotide Identity Multilocus Sequence Typing (MLST) sequence similarity to the type strain. Surprisingly, more than half of the genomes initially designated as *Pseudomonas syringae* and *Pseudomonas fluorescens* should be classified either to a previously described species or to a new genospecies. Notably, high pairwise average nucleotide identity (>95%) indicating species-level similarity was observed between *P. synxantha-P. libanensis, P. psychrotolerans*–*P. oryzihabitans*, and *P. kilonensis- P. brassicacearum*, that were previously differentiated based on conventional biochemical tests and/or genome-genome hybridization techniques.

## Introduction

The genus *Pseudomonas* is considerably diverse and consists of more than 100 characterized species to date (Mulet et al., 2013). Some species of *Pseudomonas* are well-known and characterized such as *P. aeruginosa* (Oliver et al., 2000). Some are plant-associated such as *Pseudomonas fluorescens* and *Pseudomonas syringae* and define or are present in numerous plant-microbe system (Pieterse et al., 2014). *Pseudomonas fluorescens* in general, is known for its ability to colonize plant rhizospheres and produce antimicrobial compounds targeting pathogens; thus, protecting plants from diseases (Hol et al., 2013). On the other hand, *P. syringae* is commonly associated with plant disease and has been known to invade a variety of plant species with its extracellular flagella and pili appendages. Infection with *P. syringae* can also predispose the host plant to environmental stresses such as frost damage (Maki et al., 1974).

The genetic threshold level for bacterial species definition has seen various changes in the past 20 years due to the development and availability of new bioinformatics tools and genetic resources. For example, Hagström et al. (2002) reported a threshold of 97% or lower in homology of the 16S rRNA DNA sequence to be sufficient to characterize two bacteria as different species. Later this value was raised and is now more widely accepted at 99% (Buckley and Roberts, 2007). However, 16S rRNA has also been previously suggested to be efficient at delineating bacterial strains to a genera but not for species identification (Moore et al., 1996; Anzai et al., 2000; Yamamoto et al., 2000).

Based on pairwise comparisons of amino acid identity (AAI) with cutoff of 95% such that members within one genomic cluster have AAI >95%, Jun et al. (2016) reported nine major groups corresponding to the major *Pseudomonas* species groups including seven well-described species *P. aeruginosa, P. fluorescens, P. syringae, P. putida, P. stuzeri, P. fragi, P. fusovaginae* and two mixtures of unidentified species. *P. aeruginosa* contributes to more than half of the 1,073 genomes used in the study and forms a single well delineated genomic cluster suggesting that it is well-characterized. On the contrary, 29 genomes deposited as *P. fluorescens* in public database were found in 20 different genomic clusters indicating potential mislabeling of these genomes. In addition, many suggestions for redefinition of some *Pseudomonas* species such as *P. avellanae* and *P. putida* have been raised (Jun et al., 2016).

In this study, we inferred the phylogenomic placement of 373 *Pseudomonas* genomes identified to the species level representing 79 unique species and evaluated their species validity based on *in silico* genome-genome hybridization. The re-classification of *Pseudomonas* strains based on whole genome sequences will assist future comparative genomics analysis study and more importantly highlights the need for a more robust classification of *Pseudomonas* strains in the future especially with the availability of new genomic resources.

## Materials and methods

### Datasets

Whole genome sequences of *Pseudomonas* strains with species designation excluding those of *P. aeruginosa* were downloaded from the NCBI FTP server (ftp.ncbi.nih.gov) in February, 2016. All genomes were filtered for assemblies with contig number no greater than 300 to avoid the inclusion of overly fragmented genome into the analysis. In addition, ten *Acinetobacter*, four *Cellvibrio* species and two *Azotobacter* species whole genomes were also included as the outgroup for phylogenomic analysis.

### Phylogenomic inference

Whole proteome was predicted using Prodigal V2.6.2 (Hyatt et al., 2010) and piped into PhyloPhlAn 0.99 (Segata et al., 2013) to construct a maximum likelihood tree using FastTree2 (–bionj –slownni –mlacc 2 –pseudo –spr 4 setting) (Price et al., 2010) based on the identification, alignment and concatenation of 400 universally conserved proteins (Edgar, 2004, 2010). Local branch support values were computed by FastTree2 using the Shimodaira-Hasegawa test. The tree was subsequently visualized and manually collapsed based on genospecies (Supplemental Table 1) using MEGA6 (Tamura et al., 2013).

### Average nucleotide identity (ANI) calculation

**Figure 1:**
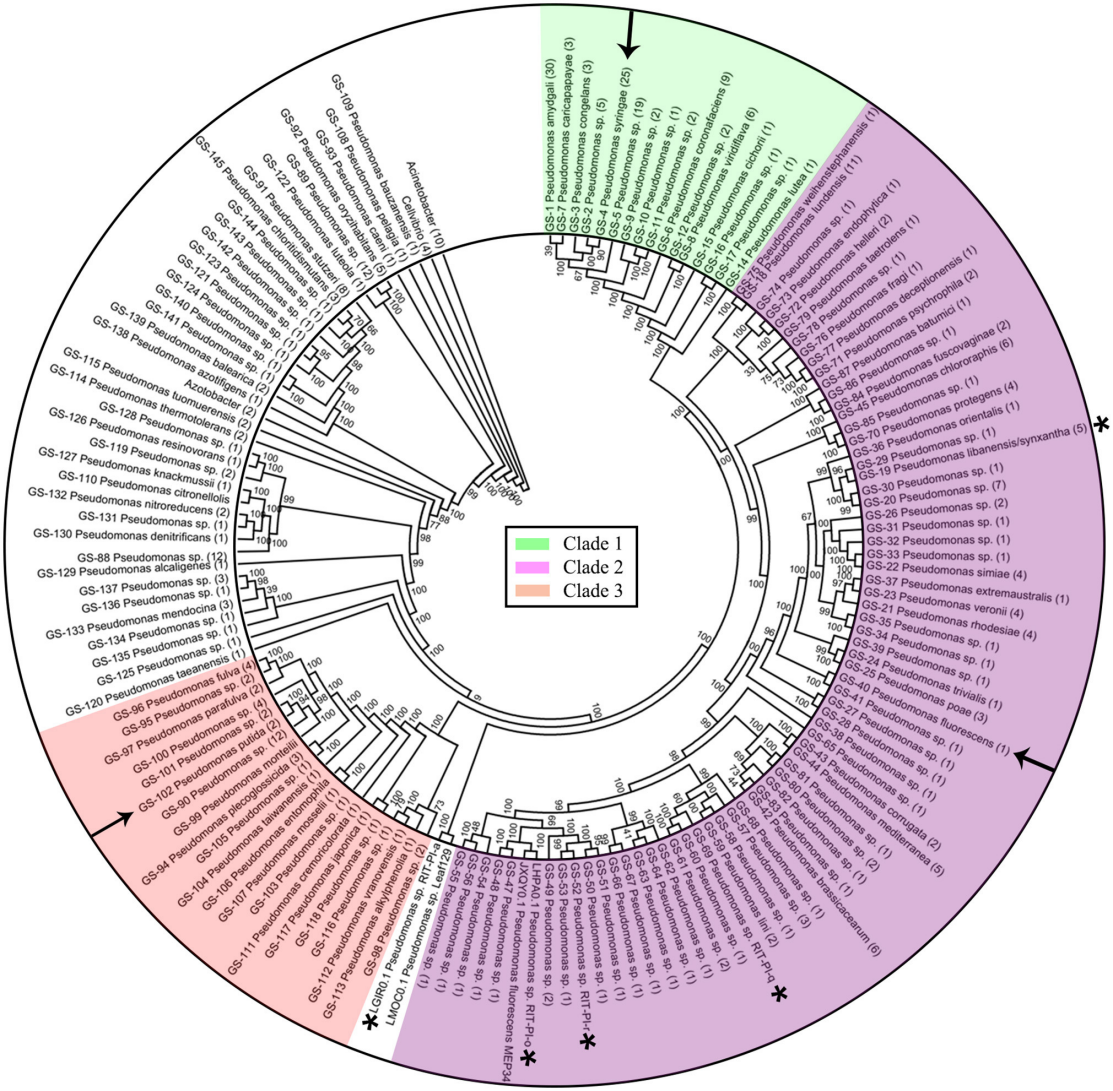
Phylogenomic tree of 373 *Pseudomonas* strains with corrected taxonomic assignments. Three major clades were colored accordingly with arrows indicating the type strains of 3 common *Pseudomonas* species e.g., *P. putida, P. fluorescens*, and *P. syringae*. Node labels indicate Shimodaira-Hasegawa(SH)-like bootstrap values in percentage. Branches classified as the same genospecies (GS) were collapsed with the number in bracket at the end of each lineage representing the number of whole genome sequences in the corresponding GS. Whole genome sequence of *P. syringae, P. fluorescens*, and *P. putida* type strains are indicated with arrows while asterisks-labeled genomes are the five *Pseudomonas* species isolated from poison ivy vine tissue.

Pairwise ANIm was calculated with Jspecies v1.2.1 using the standard MUMmer algorithm (Richter and Rosselló-Móra, 2009). To reduce computational calculation, we separated the ANIm calculation into 4 groups based on phylogenomic clustering e.g., Clades 1, 2, 3 and strains that are not part of the labeled clade (Figure 1). The matrix obtained from Jspecies for each major group was clustered and visualized using Rstudio 0.99.902, pheatmap package.

### Housekeeping gene similarity calculation

Nucleotide similarity search on housekeeping genes (*rpoB, recA, rpoD*, and/or *gyrB*) was performed with basic local alignment search tool (BLASTN) using an *e*-value cutoff of 1 × e^−10^ (Altschul et al., 1990).

## Results and discussion

### High genomic diversity among *Pseudomonas* strains

A total of 373 *Pseudomonas* genome sequences were selected for phylogenomic tree reconstruction. The genome size among *Pseudomonas* strains is highly variable, ranging from 3,022,325 bp (*P. caeni* DSM 24390) to 8,608,769 bp (*P. bauzanesis* W13Z2). Similarly, the GC content of their genomes ranges from 56.51% (*P. endophytica* BSTT44) to 67.43% (*P. syringae pv tomato* T1). Based on current classification, the most commonly deposited non-*P. aeruginosa* pseudomonad species is *P. syringae* (*N* = 67), followed by *P. fluorescens* (*N* = 62), *P. putida* (*N* = 28), *P. stuzeri* (*N* = 17), *P. amygdali* (*N* = 15), *P. psychrotolerans* (*N* = 14), *P. savastanoi* (*N* = 13), and *P. denitrificans* (*N* = 11). Out of a total of 79 species outcomes, surprisingly 43 species have only one whole genome sequence representative in the database.

Maximum likelihood tree as inferred from 400 universal conserved proteins clustered the *Pseudomonas* strains into several major clades with maximal SH-like branch support (Figure 1 and Supplemental Figure 1). Support values of some inner clades are low suggesting that shallow relationships between strains within the same genospecies are not resolved, presumably due to the lack of divergence at the amino acid level among closely related strains. A wgMLST analysis restricted to strains from closely related *Pseudomonas* species will likely identify more conserved loci which can then be used to refine their evolutionary relationships which unfortunately is beyond the scope of this study. Such wgMLST analysis can be performed using existing bioinformatic tools such as BIGSdb, Roary and PGAdb-builder (Jolley and Maiden, 2010; Page et al., 2015; Liu et al., 2016). Strikingly, several whole genome sequences labeled as the same *Pseudomonas* species were located in different major clades or distantly apart taxa (Supplemental Figure 1). For example, while *P. protegens* CHA0^T^ clustered within Clade 2, *P. protegens* 231 PPRO formed a monophyletic group with other sequences labeled as *P. denitrificans* in a distant clade. Similarly, *P. marginalis* ICMP 11289 belongs to Clade 1 and clusters with other *P. viridiflava* whereas *P. marginalis* ICMP 9505 belongs to Clade 2 (Supplemental Figure 1). The lack of taxonomic congruence as reflected by the inconsistent phylogenetic clustering among *Pseudomonas* strains with the same species designation raises suspicion about the species validity of *Pseudomonas* genomes submitted to public database.

### Disentangling the taxonomy of *Pseudomonas* using *In silico* genome-genome hybridization

Using 95% ANIm as the cutoff point for species delineation, a total of 145 genomic clusters were formed and when possible, assigned to a valid *Pseudomonas* species (Figure 1 and Supplemental Table 1). Two-hundred and seven genomes were wrongly assigned at the species level as evidence by the lack of genomic clustering with their expected type strain genome and/or low nucleotide similarity (<95%) to the type strain Multilocus Sequence Typing (MLST) sequences.

**Figure 2:**
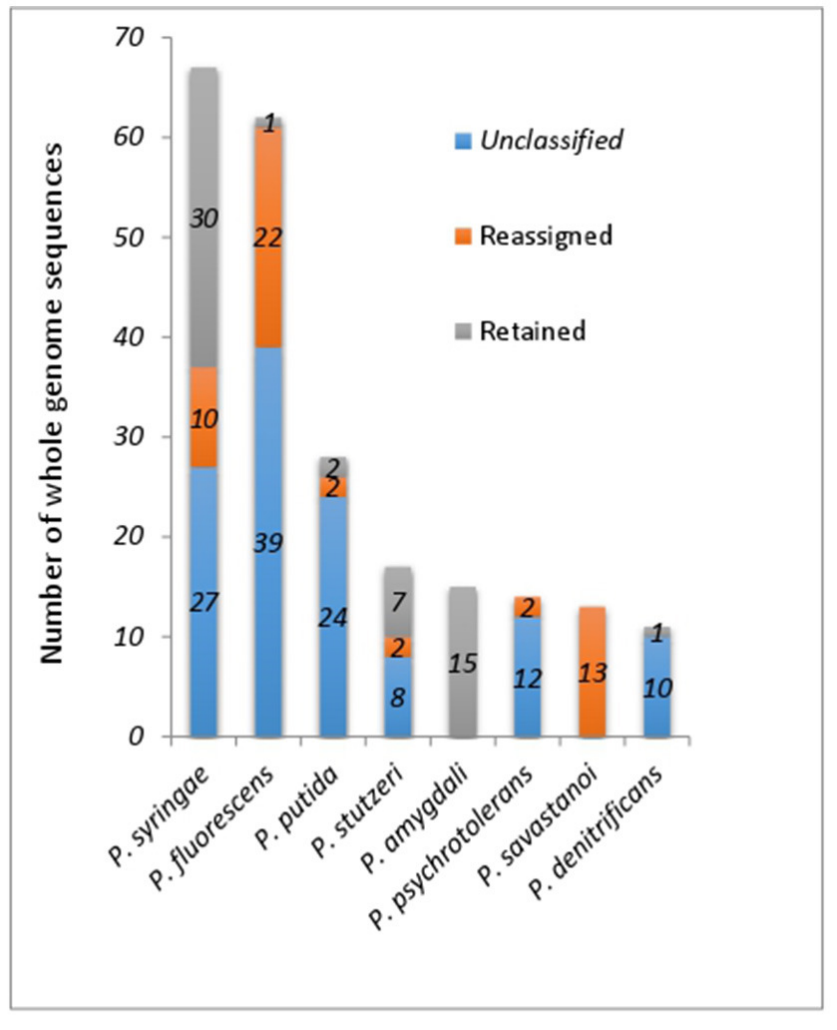
Proportion of retained, reassigned and unclassified whole genome sequences within 8 most common *Pseudomonas* species excluding *P. aeruginosa*.

All of the major species contain at least one member with incorrect species classification except for *P. amygdali* (Figure 2). Surprisingly, all 13 *P. savastanoi* genomes, majority of which originated from a single study (Mott et al., 2016) may have been misclassified as they were clustered into GS-1 containing *P. amygdali* ICMP3918^T^ (Figure 2 and Supplemental Table 1). ANIm matrix of 113 whole genome sequences within Clade 1 (Figure 1) containing a majority of *P. syringae* identified at least 14 different genomic clusters (Supplemental Figure 2). The presence of whole genome sequence for the *P. syringae* type strain KTCC 12500^T^ = DSM 10604^T^ in GS-4 suggests that only 23 out of 67 *P. syringae* genomes were correctly assigned (Supplemental Figure 2), indicating that more than 50% of the currently deposited *P. syringae* genomes should be taxonomically revised. At least 10 *P. syringae* genomes have to be assigned to other valid *Pseudomonas* species (Supplemental Table 1). For example, *P. syringae* CC1513 should be re-classified as *P. coronafaciens* given its high pairwise ANIm of 99.2% to *P. coronafaciens* LMG5060^T^.

Prior to the genome availability of *P. fluorescens* DSM 50090^T^, *P. fluorescens* strain WH6 has served as the reference genome for the taxonomic assignment of *P. fluorescens* (Duan et al., 2013; Feng et al., 2015). Unfortunately, ANIm calculation shows that strain WH6 belongs to GS-35 with a pairwise ANIm of only 87.94% to the recently available *P. fluorescens* DSM 50090^T^ genome (Supplemental Table 1 and Supplemental Figure 3). In another study, strain WH6 was also shown to be more similar to *P. azotofomans* LMG 21611^T^ than the representative species *P. fluorescens* DSM 50090^T^ using the MLSA method (Gomila et al., 2015). This finding along with our results here, suggest that the designation of *P. fluorescens* WH6 genome as the reference genome might have led to the subsequent mislabeling of newly-sequenced *Pseudomonas* genomes. As expected, out of a total of 62 whole genome sequences that were assigned as *P. fluorescens*, none of them belongs to the same genospecies as *P. fluorescens* DSM 50090^T^. By re-evaluating the species designation based on ANIm clustering against other type strains of *Pseudomonas*, 22 of the 61 wrongly identified *P. fluorescens* strains were successfully re-assigned to the correct valid species name leaving 39 pseudomonad genospecies in Clade 2 (Figure 1) unassigned at the species level.

Only 1 strain of the total 28 *P. putida* strains belongs to the same genospecies with the *P. putida* type strain NBRC 14164^T^ (GS-102) which was similarly supported by their monophyletic clustering in the phylogenomic tree (Clade 3 in Supplemental Figure 1). Some of the mislabeled *P. putida* strains might be novel species given its independent formation of genospecies e.g., GS-103 in *P. putida* MTCC5279 (Supplemental Table 1 and Supplemental Figure 4).

### Species validity of closely related *Pseudomonas* species

#### *Pseudomonas libanensis* and *Pseudomonas synxantha*

**Figure 3:**
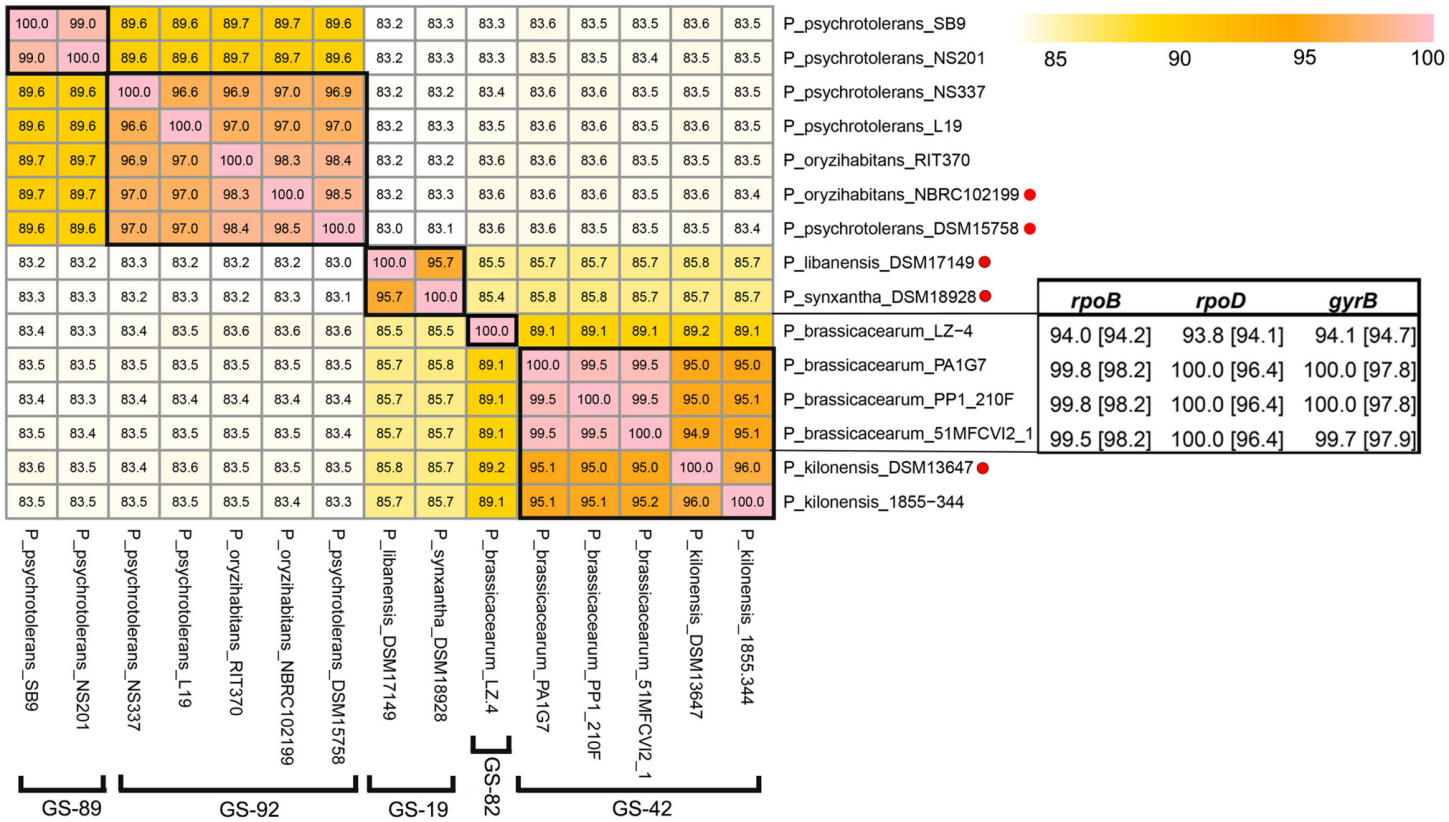
ANIm matrix of 15 *Pseudomonas* genomes from closely-related species including *P. psychrotolerans*-*P. oryzihanbitans, P. brassicacearum*-*P. kilonensis*, and *P. libanensis*-*P. synxantha*. Type strains were indicated with red dots and the genospecies corresponding to each cluster was specified in bracket. Blastn results were expressed in percentage with values in and outside the bracket show percentage identity to the housekeeping genes (*rpoB, rpoD*, and *gyrB*) of *P. kilonensis* DSM 13647^T^ and *P. brassicacearum* CIP 107059^T^, respectively. These two genomes along with whole genome of *P. psychrotolerans* DSM 15758^T^ (downloaded in April 2017) were only used to generate the ANIm matrix and were excluded from other analyses.

*Pseudomonas libanensis* DSM 17149^T^ was isolated from Lebanon spring water whereas *P. synxantha* DSM 18928^T^ was isolated from milk cream in Iowa, USA (De Vos et al., 1989). Despite a pairwise 16S rDNA similarity of up to 99.5% between *P. libanensis* DSM 17149^T^ and *P. synxantha* DSM 18928^T^, these two strains were considered as different species on the basis that strikingly high 16S rDNA similarity does not corroborate their reported low relative binding ratio of DNAs (RBR) (Dabboussi et al., 1999). The difference between *P. libanensis* and *P. synxantha* was further substantiated by other phenotypes such as levan formation and assimilation of histidine and erythritol (Dabboussi et al., 1999). Since 2005, both species have been included in the Bergey's Manual (Palleroni, 2005). However, it is worth noting that the difference in biochemical property can be due to single nucleotide polymorphism or presence/absence of horizontally transfer gene(s) that does not contribute significantly to the overall nucleotide difference at the genomic level (Carnoy and Moseley, 1997; Boddicker et al., 2002; Monk et al., 2017). Furthermore, conventional DNA-DNA hybridization (DDH) procedures are technically demanding yet error-prone and often result in different outcomes (Rosselló-Mora, 2006; Johnson and Whitman, 2007). On the other hand, digital DDH is considered a more robust, pragmatic and an accurate replacement method for conventional DDH procedures (Richter and Rosselló-Móra, 2009; Auch et al., 2010). Contrary to their low RBR result, the whole genome sequences of *P. synxantha* DSM 18928^T^ and *P. libanensis* DSM 17149^T^ exhibit a pairwise ANIm of 96.7% clustering placing them both into GS-19 (Figure 3). In other words, genomic evidence does not support the classification of *P. synxantha* and *P. libanensis* as two separate species, warranting additional taxonomic investigations.

#### *Pseudomonas brassicacearum* and *Pseudomonas kilonensis*

Similarly, *Pseudomonas brassicacearum* and *Pseudomonas kilonensis* were described as two distinct species (Sikorski et al., 2001) on the basis of substantial dissimilarity in metabolic properties and a surprisingly borderline DNA-DNA hybridization similarity for species delineation. *P. kilonensis* was isolated from agricultural soil of northern Germany and described as a distinct species from *P. brassicacearum* by 10-12 different metabolic properties with convential DNA-DNA hybridization similarity of 93% (Sikorski et al., 2001). *Pseudomonas brassicacearum* was firstly described in 2000 when these strains were isolated from the rhizoplane of *Arabidopsis thaliana* and *Brassica napus* growing on different soils (Achouak et al., 2000). Since whole genome sequence of *P. brassicacearum* type strain is not yet available, blastn was conducted against housekeeping genes (*rpoB, rpoD* and *gyrB*) of *P. brassicacearum* CFBP 11706^T^ = CIP 107059^T^ and *P. kilonensis* 520-20^T^ = DSM 13647^T^ to validate the correct identification of 3 *P. brassicacearum* genomes belonging to GS-42 (Figure 3). *P. kilonensis* DSM 13647^T^ also show strikingly high pairwise ANIm to these three *P. brassicacearum* genomes leading to the clustering of all strains to GS-42, indicating that *P. kilonensis* should be considered as the junior synonym to *P. brassicacearum* as *P. brassicacearum* was described earlier than *P. kilonensis*.

#### *Pseudomonas psychrotolerans* and *Pseudomonas oryzihabitans*

Fourteen whole genome sequences labeled as *P. psychrotolerans* were distributed into two separate genospecies: GS-89 and GS-92. The presence of both *P. oryzihabitans* NBRC 102199^T^ and *P. psychrotolerans* DSM 15758^T^ in GS-92 implies that the two species are closely-related (ANIm similarity of 98.5%). *P. psychrotolerans* strain NS337 and L19 were correctly labeled but the other 12 sequences in GS-89 including strain SB9 and NS201 were incorrectly identified (Figure 3). *P. psychrotolerans* was firstly isolated from small European ungulates (Hauser et al., 2004) whereas *P. oryzihabitans* was isolated from soil in rice paddies (Kodama et al., 1985). It is suggested that *P. psychrotolerans* can be a junior synonym of *P. oryzihabitans* for such high similarity in digital DNA-DNA hybridization.

### Taxonomic re-evaluation of *Pseudomonas* strains isolated from poison ivy (*Toxicodendron radicans*) vine tissue

With the current availability of defined genospecies within the genus *Pseudomonas*, we re-evaluated the taxonomic status of 5 *Pseudomonas* strains previously isolated from interior vine tissue of poison ivy by our group (Tran et al., 2015). Strain RIT-PI-g could be assigned to the species *Pseudomonas libanensis* forming GS-19 along with the *P. libanensis* DSM 17149^T^ corroborating our previous species assignment based on similarity to various housekeeping genes. *Pseudomonas*. sp. RIT-PI-q and *P*. sp. RIT-PI-r both belong to a single-member genospecies (GS-69 and GS-52, respectively), further underscoring the high genomic diversity of plant-associated *Pseudomonas* that remains to be explored and described with extensive taxon sampling effort. On the contrary, *Pseudomonas* sp. RIT-PI-o and *P*. sp. RIT-PI-a were placed in the same genomic cluster (GS-13) as the incorrectly labeled *P. fluorescens* MEP34 and *Pseudomonas* sp. leaf129 (Supplemental Table 1 and Supplemental Figure 5). Both *P. fluorescens* MEP34 and *P.s* sp. leaf129 were isolated from leaf of *Arabidopsis thaliana*, a popular flowering plant in North America that has been adopted as the flowering plant genetic model species (Bai et al., 2015; Perisin et al., 2015). Such similar plant origins, e.g., poision ivy vine and Arabidopsis, poses interesting links between bacterial genomic characteristics and plant-hosting capacity.

## Conclusions

The bacterial community has waited many years for the whole genome of *P. fluorescens* type strain to be sequenced (Flury et al., 2016). It is also surprising to note that whole genome sequence of some well-known *Pseudomonas* species such as *P. brassicacearum* is still unavailable despite the dramatic reduction in sequencing cost. Usually, a traditional BLASTn against 16S rRNA database was performed with varying degree of analysis depth to taxonomically assign newly-sequenced bacterial genomes which has potentially resulted in taxonomic misclassifications of several *Pseudomonas* genomes. Our study shows that in the future, a phylogenomic inference coupled with ANIm calculation could be a practical and a more reproducible method for inferring accurate genomic relatedness among *Pseudomonas* strains.

## Author contributions

PT and HG conceived the experiments. PT performed the analysis of the data. PT, HG, and MS interpret the results and contributed to the manuscript write-up.

### Conflict of interest statement

The authors declare that the research was conducted in the absence of any commercial or financial relationships that could be construed as a potential conflict of interest.

## Supplementary material

Supplemental Figure 1 Phylogenomic tree of 373 *Pseudomonas* strains with original taxonomic assignments. Three major clades were colored accordingly with arrows indicating the type strains of 3 common *Pseudomonas* species e.g., *P. putida, P. fluorescens, P. syringae*. Whole genome sequence of *P. syringae, P. fluorescens* and *P. putida* type strains are indicated with arrows while asterisks-labeled genomes are the five *Pseudomonas* species isolated from poison ivy vine tissue.

Supplemental Figure 2 Genomic clustering of Clade 1 using ANIm calculation.

Supplemental Figure 3 Genomic clustering of Clade 2 using ANIm calculation.

Supplemental Figure 4 Genomic clustering of Clade 3 using ANIm calculation.

Supplemental Figure 5 Genomic clustering of other *Pseudomonas* strains using ANIm calculation.

Supplemental Table 1 Genomic clusters of Clade 1, Clade 2, Clade 3 and other *Pseudomonas* strains with suggested assignation. Mislabeled genomes are indicated with asterisk whereas type strains are labeled “T.”
