# Supplementary figures and images for: In-silico Taxonomic Classification of 373 Genomes Reveals Species Misidentification and New Genospecies within the Genus Pseudomonas
Source: Front Microbiol. 2017 Jul 12;8:1296. doi: 10.3389/fmicb.2017.01296 (PMC5506229; doi:10.3389/fmicb.2017.01296)

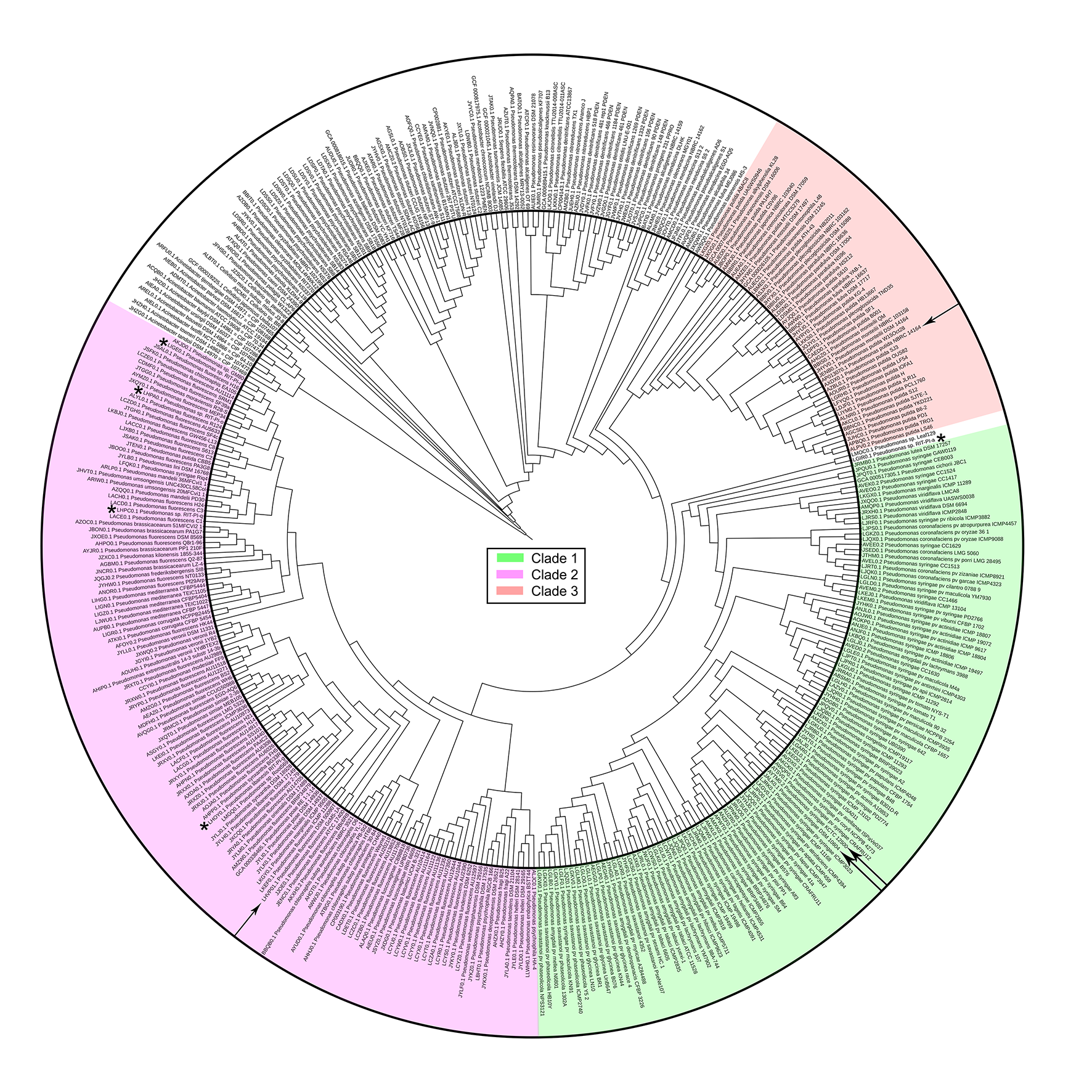

Supplement: Supplemental Figure 1 — Phylogenomic tree of 373 Pseudomonas strains with original taxonomic assignments. Three major clades were colored accordingly with arrows indicating the type strains of 3 common Pseudomonas species e.g., P. putida, P. fluorescens, P. syringae. Whole genome sequence of P. syringae, P. fluorescens and P. putida type strains are indicated with arrows while asterisks-labeled genomes are the five Pseudomonas species isolated from poison ivy vine tissue. [file Image1.TIF]

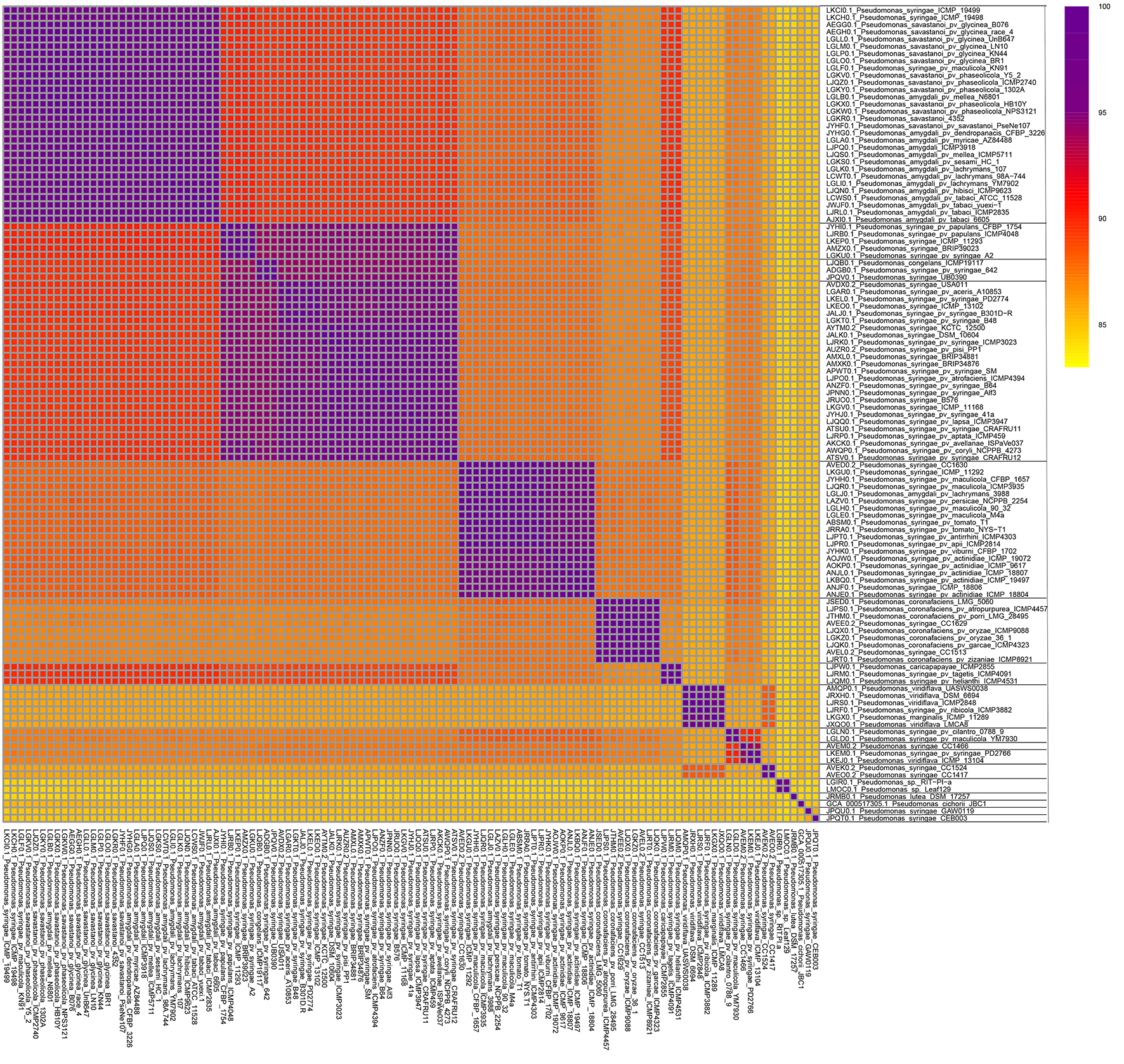

Supplement: Supplemental Figure 2 — Genomic clustering of Clade 1 using ANIm calculation. [file Image2.TIF]

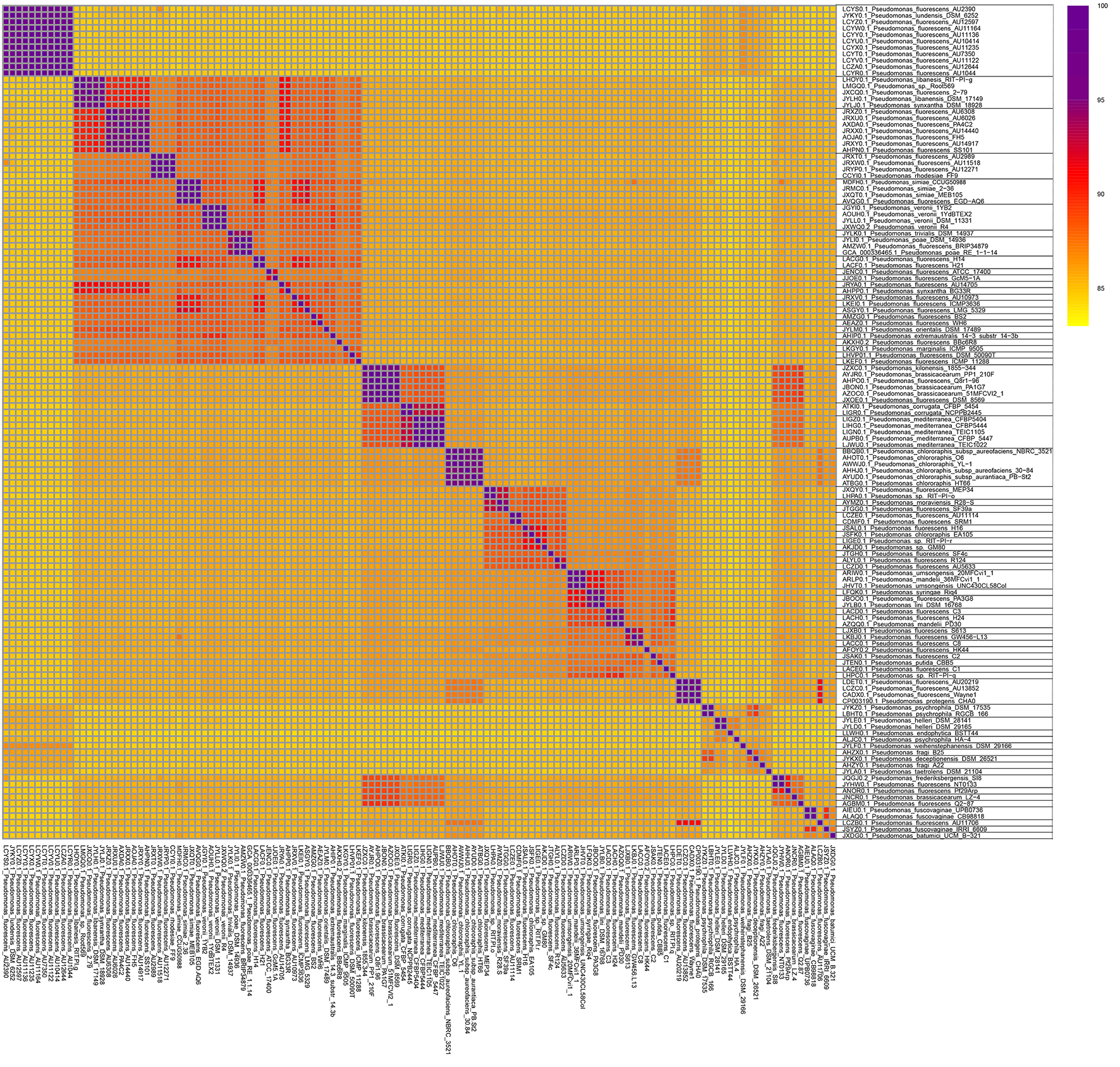

Supplement: Supplemental Figure 3 — Genomic clustering of Clade 2 using ANIm calculation. [file Image3.TIF]

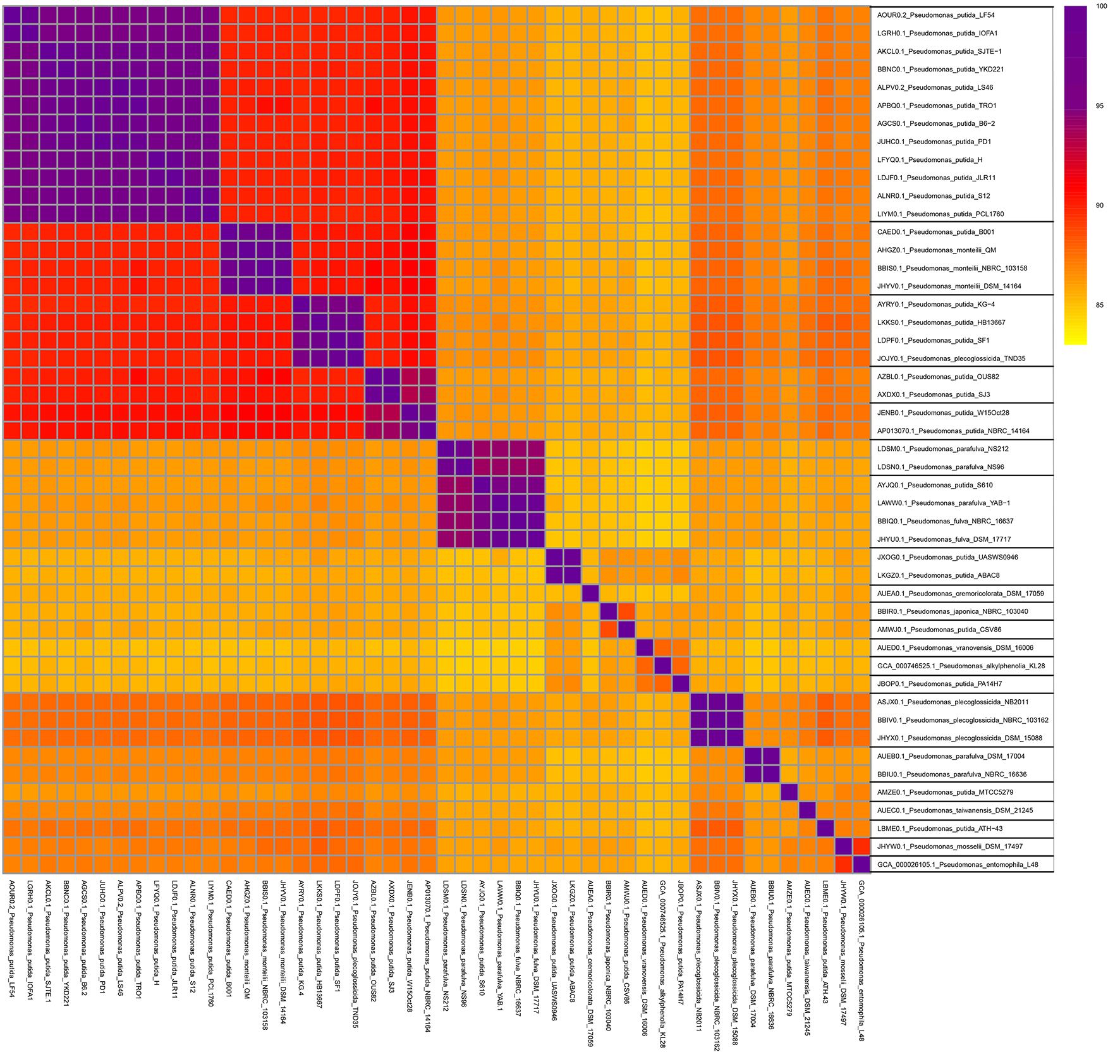

Supplement: Supplemental Figure 4 — Genomic clustering of Clade 3 using ANIm calculation. [file Image4.TIF]

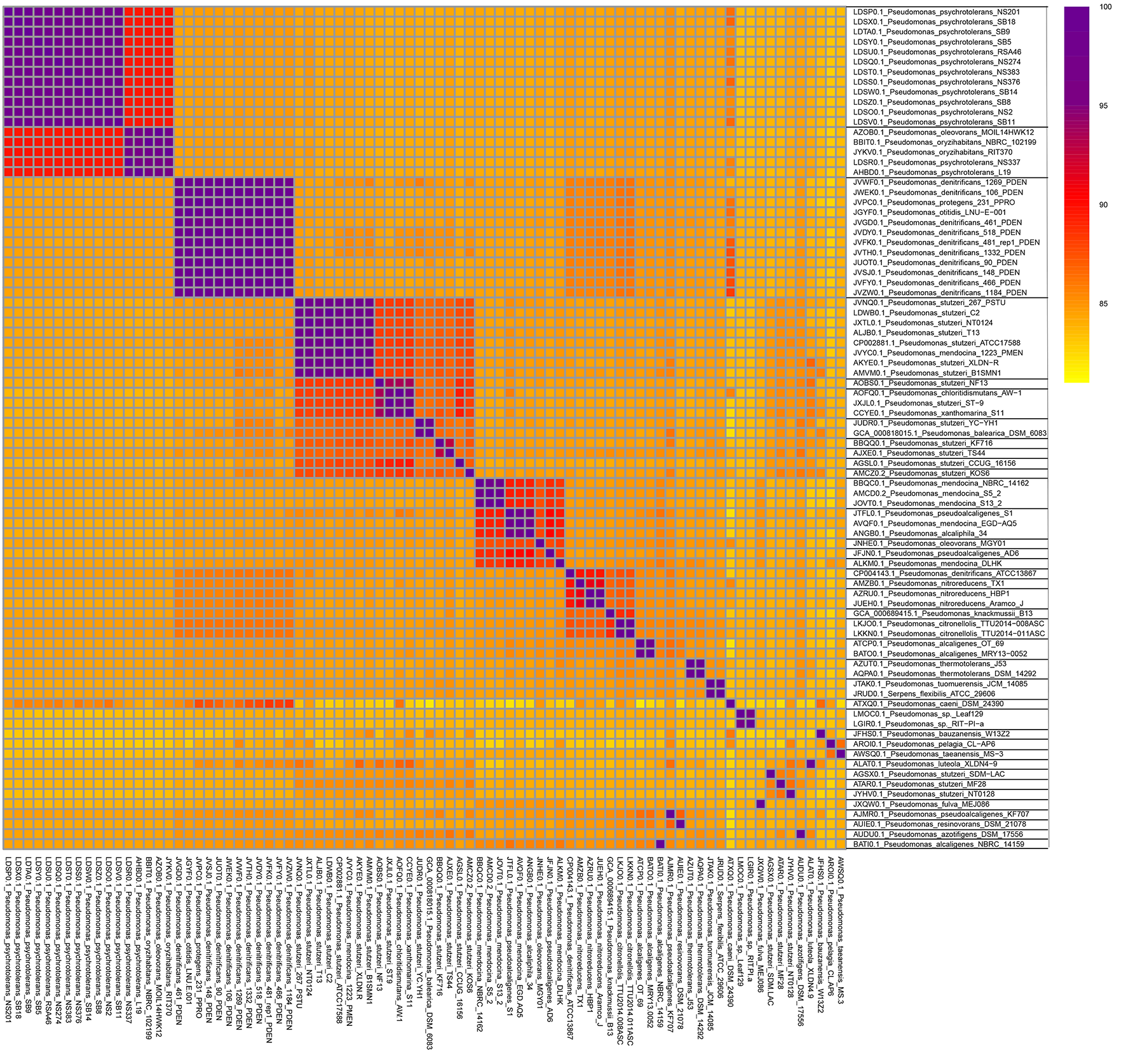

Supplement: Supplemental Figure 5 — Genomic clustering of other Pseudomonas strains using ANIm calculation. [file Image5.TIF]
