# Supplementary material for: In-silico Taxonomic Classification of 373 Genomes Reveals Species Misidentification and New Genospecies within the Genus Pseudomonas
Source: Front Microbiol. 2017 Jul 12;8:1296. doi: 10.3389/fmicb.2017.01296 (PMC5506229; doi:10.3389/fmicb.2017.01296)
Supplement: Supplemental Table 1 — Genomic clusters of Clade 1, Clade 2, Clade 3 and other Pseudomonas strains with suggested assignation. Mislabeled genomes are indicated with asterisk whereas type strains are labeled “T.” [file Table1.DOCX]

| **Genospecies** | **Name** |  | **Blast/ type strain and genes** | **Accession number** |
| --- | --- | --- | --- | --- |
| 1 – Pseudomonas amydgali | Pseudomonas syringae ICMP 19499 | * |  | LKCI0.1 |
|  | Pseudomonas syringae ICMP 19498 | * |  | LKCH0.1 |
|  | Pseudomonas savastanoi pv glycinea B076 | * |  | AEGG0.1 |
|  | Pseudomonas savastanoi pv glycinea race 4 | * |  | AEGH0.1 |
|  | Pseudomonas savastanoi pv glycinea UnB647 | * |  | LGLL0.1 |
|  | Pseudomonas savastanoi pv glycinea LN10 | * |  | LGLM0.1 |
|  | Pseudomonas savastanoi pv glycinea KN44 | * |  | LGLP0.1 |
|  | Pseudomonas savastanoi pv glycinea BR1 | * |  | LGLO0.1 |
|  | Pseudomonas syringae pv maculicola KN91 | * |  | LGLF0.1 |
|  | Pseudomonas savastanoi pv phaseolicola Y5 2 | * |  | LGKV0.1 |
|  | Pseudomonas savastanoi pv phaseolicola ICMP2740 | * |  | LJQZ0.1 |
|  | Pseudomonas savastanoi pv phaseolicola 1302A | * |  | LGKY0.1 |
|  | Pseudomonas amygdali pv mellea N6801 |  |  | LGLB0.1 |
|  | Pseudomonas savastanoi pv phaseolicola HB10Y | * |  | LGKX0.1 |
|  | Pseudomonas savastanoi pv phaseolicola NPS3121 | * |  | LGKW0.1 |
|  | Pseudomonas savastanoi 4352 | * |  | LGKR0.1 |
|  | Pseudomonas savastanoi pv savastanoi PseNe107 | * |  | JYHF0.1 |
|  | Pseudomonas amygdali pv dendropanacis CFBP 3226 |  |  | JYHG0.1 |
|  | Pseudomonas amygdali pv myricae AZ84488 |  |  | LGLA0.1 |
|  | Pseudomonas amygdali ICMP3918 | T |  | LJPQ0.1 |
|  | Pseudomonas amygdali pv mellea ICMP5711 |  |  | LJQS0.1 |
|  | Pseudomonas amygdali pv sesami HC 1 |  |  | LGKS0.1 |
|  | Pseudomonas amygdali pv lachrymans 107 |  |  | LGLK0.1 |
|  | Pseudomonas amygdali pv lachrymans 98A−744 |  |  | LCWT0.1 |
|  | Pseudomonas amygdali pv lachrymans YM7902 |  |  | LGLI0.1 |
|  | Pseudomonas amygdali pv hibisci ICMP9623 |  |  | LJQN0.1 |
|  | Pseudomonas amygdali pv tabaci ATCC 11528 |  |  | LCWS0.1 |
|  | Pseudomonas amygdali pv tabaci yuexi−1 |  |  | JWJF0.1 |
|  | Pseudomonas amygdali pv tabaci ICMP2835 |  |  | LJRL0.1 |
|  | Pseudomonas amygdali pv tabaci 6605 |  |  | AJXI0.1 |
| 2 – Pseudomonas sp. | Pseudomonas syringae pv papulans CFBP 1754 |  |  | JYHI0.1 |
|  | Pseudomonas syringae pv papulans ICMP4048 |  |  | LJRB0.1 |
|  | Pseudomonas syringae ICMP 11293 |  |  | LKEP0.1 |
|  | Pseudomonas syringae BRIP39023 |  |  | AMZX0.1 |
|  | Pseudomonas syringae pv syringae A2 |  |  | LGKU0.1 |
| 3 – Pseudomonas congelans | Pseudomonas congelans ICMP19117 | T |  | LJQB0.1 |
|  | Pseudomonas syringae pv syringae 642 | * |  | ADGB0.1 |
|  | Pseudomonas syringae UB0390 | * |  | JPQV0.1 |
| 4 – Pseudomonas syringae | Pseudomonas syringae USA011 |  |  | AVDX0.2 |
|  | Pseudomonas syringae pv aceris A10853 |  |  | LGAR0.1 |
|  | Pseudomonas syringae pv syringae PD2774 |  |  | LKEL0.1 |
|  | Pseudomonas syringae ICMP 13102 |  |  | LKEO0.1 |
|  | Pseudomonas syringae pv syringae B301D−R |  |  | JALJ0.1 |
|  | Pseudomonas syringae pv syringae B48 |  |  | LGKT0.1 |
|  | Pseudomonas syringae KCTC 12500 | T |  | AYTM0.2 |
|  | Pseudomonas syringae DSM 10604 | T |  | JALK0.1 |
|  | Pseudomonas syringae pv syringae ICMP3023 |  |  | LJRK0.1 |
|  | Pseudomonas syringae pv pisi PP1 |  |  | AUZR0.2 |
|  | Pseudomonas syringae BRIP34881 |  |  | AMXL0.1 |
|  | Pseudomonas syringae BRIP34876 |  |  | AMXK0.1 |
|  | Pseudomonas syringae pv syringae SM |  |  | APWT0.1 |
|  | Pseudomonas syringae pv atrofaciens ICMP4394 |  |  | LJPO0.1 |
|  | Pseudomonas syringae pv syringae B64 |  |  | ANZF0.1 |
|  | Pseudomonas syringae pv syringae Alf3 |  |  | JPNN0.1 |
|  | Pseudomonas syringae B576 |  |  | JRUO0.1 |
|  | Pseudomonas syringae ICMP 11168 |  |  | LKGV0.1 |
|  | Pseudomonas syringae pv syringae 41a |  |  | JYHJ0.1 |
|  | Pseudomonas syringae pv lapsa ICMP3947 |  |  | LJQQ0.1 |
|  | Pseudomonas syringae pv syringae CRAFRU11 |  |  | ATSU0.1 |
|  | Pseudomonas syringae pv aptata ICMP459 |  |  | LJRP0.1 |
|  | Pseudomonas syringae pv avellanae ISPaVe037 |  |  | AKCK0.1 |
|  | Pseudomonas syringae pv coryli NCPPB 4273 |  |  | AWQP0.1 |
|  | Pseudomonas syringae pv syringae CRAFRU12 |  |  | ATSV0.1 |
| 5 – Pseudomonas sp. | Pseudomonas syringae CC1630 | * |  | AVED0.2 |
|  | Pseudomonas syringae ICMP 11292 | * |  | LKGU0.1 |
|  | Pseudomonas syringae pv maculicola CFBP 1657 | * |  | JYHH0.1 |
|  | Pseudomonas syringae pv maculicola ICMP3935 | * |  | LJQR0.1 |
|  | Pseudomonas amygdali pv lachrymans 3988 | * |  | LGLJ0.1 |
|  | Pseudomonas syringae pv persicae NCPPB 2254 | * |  | LAZV0.1 |
|  | Pseudomonas syringae pv maculicola 90 32 | * |  | LGLH0.1 |
|  | Pseudomonas syringae pv maculicola M4a | * |  | LGLE0.1 |
|  | Pseudomonas syringae pv tomato T1 | * |  | ABSM0.1 |
|  | Pseudomonas syringae pv tomato NYS−T1 | * |  | JRRA0.1 |
|  | Pseudomonas syringae pv antirrhini ICMP4303 | * |  | LJPT0.1 |
|  | Pseudomonas syringae pv apii ICMP2814 | * |  | LJPR0.1 |
|  | Pseudomonas syringae pv viburni CFBP 1702 | * |  | JYHK0.1 |
|  | Pseudomonas syringae pv actinidiae ICMP 19072 | * |  | AOJW0.1 |
|  | Pseudomonas syringae pv actinidiae ICMP 9617 | * |  | AOKP0.1 |
|  | Pseudomonas syringae pv actinidiae ICMP 18807 | * |  | ANJL0.1 |
|  | Pseudomonas syringae pv actinidiae ICMP 19497 | * |  | LKBQ0.1 |
|  | Pseudomonas syringae ICMP 18806 | * |  | ANJF0.1 |
|  | Pseudomonas syringae pv actinidiae ICMP 18804 | * |  | ANJE0.1 |
| 6 – Pseudomonas coronafaciens | Pseudomonas coronafaciens LMG 5060 | T |  | JSED0.1 |
|  | Pseudomonas coronafaciens pv atropurpurea ICMP4457 |  |  | LJPS0.1 |
|  | Pseudomonas coronafaciens pv porri LMG 28495 |  |  | JTHM0.1 |
|  | Pseudomonas syringae CC1629 | * |  | AVEE0.2 |
|  | Pseudomonas coronafaciens pv oryzae ICMP9088 |  |  | LJQX0.1 |
|  | Pseudomonas coronafaciens pv oryzae 36 1 |  |  | LGKZ0.1 |
|  | Pseudomonas coronafaciens pv garcae ICMP4323 |  |  | LJQK0.1 |
|  | Pseudomonas syringae CC1513 | * |  | AVEL0.2 |
|  | Pseudomonas coronafaciens pv zizaniae ICMP8921 |  |  | LJRT0.1 |
| 7 – Pseudomonas caricapapayae | Pseudomonas caricapapayae ICMP2855 | T |  | LJPW0.1 |
|  | Pseudomonas syringae pv tagetis ICMP4091 | * |  | LJRM0.1 |
|  | Pseudomonas syringae pv helianthi ICMP4531 | * |  | LJQM0.1 |
| 8 – Pseudomonas viridiflava | Pseudomonas viridiflava UASWS0038 |  |  | AMQP0.1 |
|  | Pseudomonas viridiflava DSM 6694 |  |  | JRXH0.1 |
|  | Pseudomonas viridiflava ICMP2848 | T |  | LJRS0.1 |
|  | Pseudomonas syringae pv ribicola ICMP3882 |  |  | LJRF0.1 |
|  | Pseudomonas marginalis ICMP 11289 | * |  | LKGX0.1 |
|  | Pseudomonas viridiflava LMCA8 |  |  | JXQO0.1 |
| 9 – Pseudomonas sp. | Pseudomonas syringae pv cilantro 0788 9 | * |  | LGLN0.1 |
|  | Pseudomonas syringae pv maculicola YM7930 | * |  | LGLD0.1 |
| 10 – Pseudomonas sp. | Pseudomonas syringae CC1466 | * |  | AVEM0.2 |
| 11 – Pseudomonas sp. | Pseudomonas syringae pv syringae PD2766 | * |  | LKEM0.1 |
|  | Pseudomonas viridiflava ICMP 13104 | * |  | LKEJ0.1 |
| 12 – Pseudomonas sp. | Pseudomonas syringae CC1524 | * |  | AVEK0.2 |
|  | Pseudomonas syringae CC1417 | * |  | AVEO0.2 |
| 13 – Pseudomonas sp. | Pseudomonas sp. RIT−PI−a |  |  | LGIR0.1 |
|  | Pseudomonas sp. Leaf129 |  |  | LMOC0.1 |
| 14 – Pseudomonas lutea | Pseudomonas lutea DSM 17257 | T |  | JRMB0.1 |
| 15 – Pseudomonas cichorii | Pseudomonas cichorii JBC1 |  | 100%/P. cichorii NCPPB 943T gyrB, rpoB, recA | GCA_000517305.1 |
| 16 – Pseudomonas sp. | Pseudomonas syringae GAW0119 | * |  | JPQU0.1 |
| 17 – Pseudomonas sp. | Pseudomonas syringae CEB003 | * |  | JPQT0.1 |
| 18 – Pseudomonas lundensis | Pseudomonas fluorescens AU2390 | * |  | LCYS0.1 |
|  | Pseudomonas lundensis DSM 6252 | T |  | JYKY0.1 |
|  | Pseudomonas fluorescens AU12597 | * |  | LCYZ0.1 |
|  | Pseudomonas fluorescens AU11164 | * |  | LCYW0.1 |
|  | Pseudomonas fluorescens AU11136 | * |  | LCYY0.1 |
|  | Pseudomonas fluorescens AU10414 | * |  | LCYU0.1 |
|  | Pseudomonas fluorescens AU11235 | * |  | LCYX0.1 |
|  | Pseudomonas fluorescens AU7350 | * |  | LCYT0.1 |
|  | Pseudomonas fluorescens AU11122 | * |  | LCYV0.1 |
|  | Pseudomonas fluorescens AU12644 | * |  | LCZA0.1 |
|  | Pseudomonas fluorescens AU1044 | * |  | LCYR0.1 |
| 19 – Pseudomonas libanensis/synxantha | Pseudomonas libanesis RIT−PI−g |  |  | LHOY0.1 |
|  | Pseudomonas sp. Root569 |  |  | LMGQ0.1 |
|  | Pseudomonas fluorescens 2−79 |  |  | JXCQ0.1 |
|  | Pseudomonas libanensis DSM 17149 | T |  | JYLH0.1 |
|  | Pseudomonas synxantha DSM 18928 | T |  | JYLJ0.1 |
| 20 – Pseudomonas sp. | Pseudomonas fluorescens AU6308 | * |  | JRXZ0.1 |
|  | Pseudomonas fluorescens AU6026 | * |  | JRXU0.1 |
|  | Pseudomonas fluorescens PA4C2 | * |  | AXDA0.1 |
|  | Pseudomonas fluorescens AU14440 | * |  | JRXX0.1 |
|  | Pseudomonas fluorescens FH5 | * |  | AOJA0.1 |
|  | Pseudomonas fluorescens AU14917 | * |  | JRXY0.1 |
|  | Pseudomonas fluorescens SS101 | * |  | AHPN0.1 |
| 21 – Pseudomonas rhodesiae | Pseudomonas fluorescens AU2989 | * |  | JRXT0.1 |
|  | Pseudomonas fluorescens AU11518 | * |  | JRXW0.1 |
|  | Pseudomonas fluorescens AU12271 | * |  | JRYP0.1 |
|  | Pseudomonas rhodesiae FF9 |  | >98%/P. rhodesiae DSM 14020TgyrB, rpoD, recA | CCYI0.1 |
| 22 – Pseudomonas simiae | Pseudomonas simiae CCUG50988 | T |  | MDFH0.1 |
|  | Pseudomonas simiae 2−36 |  |  | JRMC0.1 |
|  | Pseudomonas simiae MEB105 |  |  | JXQT0.1 |
|  | Pseudomonas fluorescens EGD−AQ6 | * |  | AVQG0.1 |
| 23 – Pseudomonas veronii | Pseudomonas veronii 1YB2 |  |  | JGYI0.1 |
|  | Pseudomonas veronii 1YdBTEX2 |  |  | AOUH0.1 |
|  | Pseudomonas veronii DSM 11331 | T |  | JYLL0.1 |
|  | Pseudomonas veronii R4 |  |  | JXWQ0.2 |
| 24 – Pseudomonas trivivalis | Pseudomonas trivialis DSM 14937 | T |  | JYLK0.1 |
| 25 – Pseudomonas poae | Pseudomonas poae DSM 14936 | T |  | JYLI0.1 |
|  | Pseudomonas fluorescens BRIP34879 | * |  | AMZW0.1 |
|  | Pseudomonas poae RE 1−1−14 |  |  | GCA_000336465.1 |
| 26 – Pseudomonas sp. | Pseudomonas fluorescens H14 | * |  | LACG0.1 |
|  | Pseudomonas fluorescens H21 | * |  | LACF0.1 |
| 27 – Pseudomonas sp. | Pseudomonas fluorescens ATCC 17400 | * |  | JENC0.1 |
| 28 – Pseudomonas sp. | Pseudomonas fluorescens GcM5−1A | * |  | JJOE0.1 |
| 29 – Pseudomonas sp. | Pseudomonas fluorescens AU14705 | * |  | JRYA0.1 |
| 30 – Pseudomonas sp. | Pseudomonas synxantha BG33R | * |  | AHPP0.1 |
| 31 – Pseudomonas sp. | Pseudomonas fluorescens AU10973 | * |  | JRXV0.1 |
| 32 – Pseudomonas sp. | Pseudomonas fluorescens ICMP3636 | * |  | LKEI0.1 |
| 33 – Pseudomonas sp. | Pseudomonas fluorescens LMG 5329 | * |  | ASGY0.1 |
| 34 – Pseudomonas sp. | Pseudomonas fluorescens BS2 | * |  | AMZG0.1 |
| 35 – Pseudomonas sp. | Pseudomonas fluorescens WH6 | * |  | AEAZ0.1 |
| 36 – Pseudomonas orientalis | Pseudomonas orientalis DSM 17489 | T |  | JYLM0.1 |
| 37 – Pseudomonas extremaustralis | Pseudomonas extremaustralis 14−3 substr 14−3b |  | 100%/P. extremaustralis DSM 17835T rpoD, gyrB, recA | AHIP0.1 |
| 38 – Pseudomonas sp. | Pseudomonas fluorescens BBc6R8 | * |  | AKXH0.2 |
| 39 – Pseudomonas sp. | Pseudomonas marginalis ICMP 9505 | * |  | LKGY0.1 |
| 40 – Pseudomonas fluorescens | Pseudomonas fluorescens DSM 50090T | T |  | LHVP01.1 |
| 41 – Pseudomonas sp. | Pseudomonas fluorescens ICMP 11288 |  |  | LKEF0.1 |
| 42 – Pseudomonas brassicacearum/kilonensis | Pseudomonas kilonensis 1855−344 |  |  | JZXC0.1 |
|  | Pseudomonas brassicacearum PP1 210F |  |  | AYJR0.1 |
|  | Pseudomonas fluorescens Q8r1−96 | * |  | AHPO0.1 |
|  | Pseudomonas brassicacearum PA1G7 |  |  | JBON0.1 |
|  | Pseudomonas brassicacearum 51MFCVI2 1 |  |  | AZOC0.1 |
|  | Pseudomonas fluorescens DSM 8569 | * |  | JXOE0.1 |
| 43 – Pseudomonas corrugata | Pseudomonas corrugata CFBP 5454 |  |  | ATKI0.1 |
|  | Pseudomonas corrugata NCPPB2445 | T |  | LIGR0.1 |
| 44 – Pseudomonas mediterranea | Pseudomonas mediterranea CFBP5404 |  |  | LIGZ0.1 |
|  | Pseudomonas mediterranea CFBP5444 |  |  | LIHG0.1 |
|  | Pseudomonas mediterranea TEIC1105 |  |  | LIGN0.1 |
|  | Pseudomonas mediterranea CFBP 5447 | T |  | AUPB0.1 |
|  | Pseudomonas mediterranea TEIC1022 |  |  | LJWU0.1 |
| 45 – Pseudomonas chlororaphis | Pseudomonas chlororaphis subsp aureofaciens NBRC 3521 |  | >98%/P. chlororaphis LMG 245T rpoD  >98%/P. chlororaphis ATCC 1385T recA, rpoB, gyrB | BBQB0.1 |
|  | Pseudomonas chlororaphis O6 |  |  | AHOT0.1 |
|  | Pseudomonas chlororaphis YL−1 |  |  | AWWJ0.1 |
|  | Pseudomonas chlororaphis subsp aureofaciens 30−84 |  |  | AHHJ0.1 |
|  | Pseudomonas chlororaphis subsp aurantiaca PB−St2 |  |  | AYUD0.1 |
|  | Pseudomonas chlororaphis HT66 |  |  | ATBG0.1 |
| 46 – Pseudomonas sp. | Pseudomonas fluorescens MEP34 | * |  | JXQY0.1 |
|  | Pseudomonas sp. RIT−PI−o |  |  | LHPA0.1 |
| 47 – Pseudomonas sp. | Pseudomonas moraviensis R28−S | * | <95%/P. moraviensis DSM 16007T | AYMZ0.1 |
| 48 – Pseudomonas sp. | Pseudomonas fluorescens SF39a | * |  | JTGG0.1 |
| 49 – Pseudomonas sp. | Pseudomonas fluorescens AU11114 | * |  | LCZE0.1 |
|  | Pseudomonas fluorescens SRM1 | * |  | CDMF0.1 |
| 50 – Pseudomonas sp. | Pseudomonas fluorescens H16 | * |  | JSAL0.1 |
| 51 – Pseudomonas sp. | Pseudomonas chlororaphis EA105 | * | <90%/P. chlororaphis LMG 245T rpoD and P. chlororaphis ATCC 13985T recA, rpoB, gyrB | JSFK0.1 |
| 52 – Pseudomonas sp. | Pseudomonas sp. RIT−PI−r |  |  | LIGE0.1 |
| 53 – Pseudomonas sp. | Pseudomonas sp. GM80 |  |  | AKJD0.1 |
| 54 – Pseudomonas sp. | Pseudomonas fluorescens SF4c | * |  | JTGH0.1 |
| 55 – Pseudomonas sp. | Pseudomonas fluorescens R124 | * |  | ALYL0.1 |
| 56 – Pseudomonas sp. | Pseudomonas fluorescens AU5633 | * |  | LCZD0.1 |
| 57 – Pseudomonas sp. | Pseudomonas umsongensis 20MFCvi1 1 | * | <96%/P. umsongensis LMG 21317T rpoB  <93%/P. umsongensis LMG 21317T recA, rpoD, gyrB | ARIW0.1 |
|  | Pseudomonas umsongensis UNC430CL58Col | * |  | ARLP0.1 |
|  | Pseudomonas mandelii 36MFCvi1 | * | <93%/P. mandelii DSM 17967T gyrB | JHVT0.1 |
| 58 – Pseudomonas sp. | Pseudomonas syringae Riq4 | * |  | LFQK0.1 |
| 59 – Pseudomonas lini | Pseudomonas fluorescens PA3G8 | * |  | JBOO0.1 |
|  | Pseudomonas lini DSM 16768 | T |  | JYLB0.1 |
| 60 – Pseudomonas sp. | Pseudomonas fluorescens C3 | * |  | LACD0.1 |
| 61 – Pseudomonas sp. | Pseudomonas fluorescens H24 | * |  | LACH0.1 |
|  | Pseudomonas mandelii PD30 | * | 96%/P. mandelii DSM 17967T gyrB | AZQQ0.1 |
| 62 – Pseudomonas sp. | Pseudomonas fluorescens S613 | * |  | LJXB0.1 |
| 63 – Pseudomonas sp. | Pseudomonas fluorescens GW456−L13 | * |  | LKBJ0.1 |
| 64 – Pseudomonas sp. | Pseudomonas fluorescens C8 | * |  | LACC0.1 |
| 65 – Pseudomonas sp. | Pseudomonas fluorescens HK44 | * |  | AFOY0.2 |
| 66 – Pseudomonas sp. | Pseudomonas fluorescens C2 | * |  | JSAK0.1 |
| 67 – Pseudomonas sp. | Pseudomonas putida CBB5 | * |  | JTEN0.1 |
| 68 – Pseudomonas sp. | Pseudomonas fluorescens C1 | * |  | LACE0.1 |
| 69 – Pseudomonas sp. | Pseudomonas sp. RIT−PI−q |  |  | LHPC0.1 |
| 70 – Pseudomonas protegens | Pseudomonas fluorescens AU20219 | * |  | LDET0.1 |
|  | Pseudomonas fluorescens AU13852 | * |  | LCZC0.1 |
|  | Pseudomonas fluorescens Wayne1 | * |  | CADX0.1 |
|  | Pseudomonas protegens CHA0 | T |  | CP003190.1 |
| 71 – Pseudomonas psychrophila | Pseudomonas psychrophila DSM 17535 | T |  | JYKZ0.1 |
|  | Pseudomonas psychrophila RGCB 166 |  |  | LBHT0.1 |
| 72 – Pseudomonas helleri | Pseudomonas helleri DSM 28141 |  |  | JYLE0.1 |
|  | Pseudomonas helleri DSM 29165 | T |  | JYLD0.1 |
| 73 – Pseudomonas endophytica | Pseudomonas endophytica BSTT44 | T |  | LLWH0.1 |
| 74 – Pseudomonas sp. | Pseudomonas psychrophila HA−4 | * |  | ALJC0.1 |
| 75 – Pseudomonas weihenstephanensis | Pseudomonas weihenstephanensis DSM 29166 | T |  | JYLF0.1 |
| 76 – Pseudomonas fragi | Pseudomonas fragi B25 |  | 100%/P. fragi CECT 446T rpoB, recA, gyrB | AHZX0.1 |
| 77 – Pseudomonas deceptionensis | Pseudomonas deceptionensis DSM 26521 | T |  | JYKX0.1 |
| 78 – Pseudomonas sp. | Pseudomonas fragi A22 | * | 95%/P. fragi CECT 446T rpoB  89%/P. fragi CECT 446T gyrB | AHZY0.1 |
| 79 – Pseudomonas taetrolens | Pseudomonas taetrolens DSM 21104 | T |  | JYLA0.1 |
| 80 – Pseudomonas sp. | Pseudomonas frederiksbergensis SI8 | * | <90%/P.frederiksbergensis DSM 13022T rpoD, gyrB | JQGJ0.2 |
|  | Pseudomonas fluorescens NT0133 | * |  | JYHW0.1 |
| 81 – Pseudomonas sp. | Pseudomonas fluorescens Pf29Arp | * |  | ANOR0.1 |
| 82 – Pseudomonas sp. | Pseudomonas brassicacearum LZ−4 | * |  | JNCR0.1 |
| 83 – Pseudomonas sp. | Pseudomonas fluorescens Q2−87 | * |  | AGBM0.1 |
| 84 – Pseudomonas fuscovaginae | Pseudomonas fuscovaginae UPB0736 |  | >98%/P. fuscovaginae LMG 2158T gyrB, rpoD, rpoB | AIEU0.1 |
|  | Pseudomonas fuscovaginae CB98818 |  |  | ALAQ0.1 |
| 85 – Pseudomonas sp. | Pseudomonas fluorescens AU11706 | * |  | LCZB0.1 |
| 86 – Pseudomonas sp. | Pseudomonas fuscovaginae IRRI 6609 | * | <95%/P. fuscovaginae LMG 2158T gyrB, rpoD, rpoB | JSYZ0.1 |
| 87 – Pseudomonas batumici | Pseudomonas batumici UCM B−321 | T |  | JXDG0.1 |
| 88 – Pseudomonas sp. | Pseudomonas denitrificans 1269 PDEN | * |  | JVWF0.1 |
|  | Pseudomonas denitrificans 518 PDEN | * |  | JVDY0.1 |
|  | Pseudomonas denitrificans 481 rep1 PDEN | * |  | JVFK0.1 |
|  | Pseudomonas denitrificans 1332 PDEN | * |  | JVTH0.1 |
|  | Pseudomonas denitrificans 1184 PDEN | * |  | JVZW0.1 |
|  | Pseudomonas denitrificans 461 PDEN | * |  | JVGD0.1 |
|  | Pseudomonas denitrificans 466 PDEN | * |  | JVFY0.1 |
|  | Pseudomonas denitrificans 106 PDEN | * |  | JWEK0.1 |
|  | Pseudomonas protegens 231 PPRO | * |  | JVPC0.1 |
|  | Pseudomonas otitidis LNU−E−001 | * | <89%/P. otitidis DSM 17224T rpoB, rpoD, gyrB | JGYF0.1 |
|  | Pseudomonas denitrificans 90 PDEN | * |  | JUOT0.1 |
|  | Pseudomonas denitrificans 148 PDEN | * |  | JVSJ0.1 |
| 89 – Pseudomonas sp. | Pseudomonas psychrotolerans SB11 | * | <96%/P. psychrotolerans LMG 21977T | LDSV0.1 |
|  | Pseudomonas psychrotolerans NS201 | * |  | LDSP0.1 |
|  | Pseudomonas psychrotolerans SB9 | * |  | LDTA0.1 |
|  | Pseudomonas psychrotolerans SB18 | * |  | LDSX0.1 |
|  | Pseudomonas psychrotolerans NS2 | * |  | LDSO0.1 |
|  | Pseudomonas psychrotolerans NS376 | * |  | LDSS0.1 |
|  | Pseudomonas psychrotolerans SB14 | * |  | LDSW0.1 |
|  | Pseudomonas psychrotolerans SB8 | * |  | LDSZ0.1 |
|  | Pseudomonas psychrotolerans SB5 | * |  | LDSY0.1 |
|  | Pseudomonas psychrotolerans RSA46 | * |  | LDSU0.1 |
|  | Pseudomonas psychrotolerans NS274 | * |  | LDSQ0.1 |
|  | Pseudomonas psychrotolerans NS383 | * |  | LDST0.1 |
| 90 – Pseudomonas sp. | Pseudomonas putida LF54 | * |  | AOUR0.2 |
|  | Pseudomonas putida IOFA1 | * |  | LGRH0.1 |
|  | Pseudomonas putida PCL1760 | * |  | LIYM0.1 |
|  | Pseudomonas putida S12 | * |  | ALNR0.1 |
|  | Pseudomonas putida H | * |  | LFYQ0.1 |
|  | Pseudomonas putida JLR11 | * |  | LDJF0.1 |
|  | Pseudomonas putida B6−2 | * |  | AGCS0.1 |
|  | Pseudomonas putida TRO1 | * |  | APBQ0.1 |
|  | Pseudomonas putida LS46 | * |  | ALPV0.2 |
|  | Pseudomonas putida PD1 | * |  | JUHC0.1 |
|  | Pseudomonas putida SJTE−1 | * |  | AKCL0.1 |
|  | Pseudomonas putida YKD221 | * |  | BBNC0.1 |
| 91 – Pseudomonas stutzeri | Pseudomonas stutzeri B1SMN1 |  |  | AMVM0.1 |
|  | Pseudomonas stutzeri XLDN−R |  |  | AKYE0.1 |
|  | Pseudomonas stutzeri ATCC17588 | T |  | CP002881.1 |
|  | Pseudomonas mendocina 1223 PMEN | * |  | JVYC0.1 |
|  | Pseudomonas stutzeri 267 PSTU |  |  | JVNQ0.1 |
|  | Pseudomonas stutzeri C2 |  |  | LDWB0.1 |
|  | Pseudomonas stutzeri T13 |  |  | ALJB0.1 |
|  | Pseudomonas stutzeri NT0124 |  |  | JXTL0.1 |
| 92 – Pseudomonas oryzihabitans | Pseudomonas psychrotolerans NS337 | * | 98%/P. psychrotolerans LMG 21977T | LDSR0.1 |
|  | Pseudomonas psychrotolerans L19 | * |  | AHBD0.1 |
|  | Pseudomonas oleovorans MOIL14HWK12 | * |  | AZOB0.1 |
|  | Pseudomonas oryzihabitans RIT370 |  |  | JYKV0.1 |
|  | Pseudomonas oryzihabitans NBRC 102199 | T |  | BBIT0.1 |
| 93 – Pseudomonas caeni | Pseudomonas caeni DSM 24390 | T |  | ATXQ0.1 |
| 94 – Pseudomonas plecoglossicida | Pseudomonas plecoglossicida DSM 15088 |  |  | JHYX0.1 |
|  | Pseudomonas plecoglossicida NBRC 103162 | T |  | BBIV0.1 |
|  | Pseudomonas plecoglossicida NB2011 |  |  | ASJX0.1 |
| 95 – Pseudomonas sp. | Pseudomonas parafulva NS96 | * |  | LDSN0.1 |
|  | Pseudomonas parafulva NS212 | * |  | LDSM0.1 |
| 96 – Pseudomonas fulva | Pseudomonas putida S610 | * |  | AYJQ0.1 |
|  | Pseudomonas parafulva YAB−1 | * |  | LAWW0.1 |
|  | Pseudomonas fulva NBRC 16637 |  |  | BBIQ0.1 |
|  | Pseudomonas fulva DSM 17717 | T |  | JHYU0.1 |
| 97 – Pseudomonas parafulva | Pseudomonas parafulva DSM 17004 | T |  | AUEB0.1 |
|  | Pseudomonas parafulva NBRC 16636 | T |  | BBIU0.1 |
| 98 – Pseudomonas sp. | Pseudomonas putida ABAC8 | * |  | LKGZ0.1 |
|  | Pseudomonas putida UASWS0946 | * |  | JXOG0.1 |
| 99 – Pseudomonas monteilii | Pseudomonas monteilii NBRC 103158 | T |  | BBIS0.1 |
|  | Pseudomonas monteilii DSM 14164 | T |  | JHYV0.1 |
|  | Pseudomonas putida B001 | * |  | CAED0.1 |
|  | Pseudomonas monteilii QM |  |  | AHGZ0.1 |
| 100 – Pseudomonas sp. | Pseudomonas putida KG−4 | * |  | AYRY0.1 |
|  | Pseudomonas putida HB13667 | * |  | LKKS0.1 |
|  | Pseudomonas plecoglossicida TND35 | * |  | JOJY0.1 |
|  | Pseudomonas putida SF1 | * |  | LDPF0.1 |
| 101 – Pseudomonas sp. | Pseudomonas putida SJ3 | * |  | AXDX0.1 |
|  | Pseudomonas putida OUS82 | * |  | AZBL0.1 |
| 102 – Pseudomonas putida | Pseudomonas putida NBRC 14164 | T |  | AP013070.1 |
|  | Pseudomonas putida W15Oct28 |  |  | JENB0.1 |
| 103 – Pseudomonas sp. | Pseudomonas putida MTCC5279 | * |  | AMZE0.1 |
| 104 – Pseudomonas taiwanensis | Pseudomonas taiwanensis DSM 21245 | T |  | AUEC0.1 |
| 105 – Pseudomonas putida | Pseudomonas putida ATH−43 | * |  | LBME0.1 |
| 106 – Pseudomonas entomophila | Pseudomonas entomophila L48 | T |  | GCA 000026105.1 |
| 107 – Pseudomonas mosselii | Pseudomonas mosselii DSM 17497 | T |  | JHYW0.1 |
| 108 – Pseudomonas pelagia | Pseudomonas pelagia CL−AP6 | T |  | AROI0.1 |
| 109 – Pseudomonas bauzanensis | Pseudomonas bauzanensis W13Z2 |  | >98%/P. bauzanensis DSM 22558T rpoD, rpoB, gyrB | JFHS0.1 |
| 110 – Pseudomonas citronellolis | Pseudomonas citronellolis TTU2014−008ASC |  | >98%/P. citronellolis CIP 104381T rpoB, oprL, oprI | LKJO0.1 |
|  | Pseudomonas citronellolis TTU2014−011ASC |  |  | LKKN0.1 |
| 111 – Pseudomonas cremoricolorata | Pseudomonas cremoricolorata DSM 17059 | T |  | AUEA0.1 |
| 112 – Pseudomonas vranovensis | Pseudomonas vranovensis DSM 16006 | T |  | AUED0.1 |
| 113 – Pseudomonas alkylphenolia | Pseudomonas alkylphenolia KL28 | T |  | GCA 000746525.1 |
| 114 – Pseudomonas thermotolerans | Pseudomonas thermotolerans DSM 14292 | T |  | AQPA0.1 |
|  | Pseudomonas thermotolerans J53 |  |  | AZUT0.1 |
| 115 – Pseudomonas tuomuerensis | Pseudomonas tuomuerensis JCM 14085 | T |  | JTAK0.1 |
|  | Serpens flexibilis ATCC 29606 |  |  | JRUD0.1 |
| 116 – Pseudomonas sp. | Pseudomonas putida PA14H7 | * |  | JBOP0.1 |
| 117 – Pseudomonas japonica | Pseudomonas japonica NBRC 103040 | T |  | BBIR0.1 |
| 118 – Pseudomonas sp. | Pseudomonas putida CSV86 | * |  | AMWJ0.1 |
| 119 – Pseudomonas sp. | Pseudomonas alcaligenes MRY13−0052 | * |  | BATO0.1 |
|  | Pseudomonas alcaligenes OT 69 | * |  | ATCP0.1 |
| 120 – Pseudomonas taeanensis | Pseudomonas taeanensis MS−3 | T |  | AWSQ0.1 |
| 121 – Pseudomonas sp. | Pseudomonas stutzeri NT0128 | * |  | JYHV0.1 |
| 122 – Pseudomonas luteola | Pseudomonas luteola XLDN4−9 |  | >98%/P. luteola CIP 102995T rpoB, rpoD, gyrB | ALAT0.1 |
| 123 - Pseudomonas sp. | Pseudomonas stutzeri SDM−LAC | * |  | AGSX0.1 |
| 124 - Pseudomonas sp. | Pseudomonas stutzeri MF28 | * |  | ATAR0.1 |
| 125 - Pseudomonas sp. | Pseudomonas fulva MEJ086 | * |  | JXQW0.1 |
| 126 – Pseudomonas resinovorans | Pseudomonas resinovorans DSM 21078 | T |  | AUIE0.1 |
| 127 - Pseudomonas knackmussii | Pseudomonas knackmussii B13 | T |  | GCA 000689415.1 |
| 128 – Pseudomonas sp. | Pseudomonas pseudoalcaligenes KF707 | * | 94%/P. pseudoalcaligenes LMG 1225T rpoB  90%/P. pseudoalcaligenes IFO 1416T | AJMR0.1 |
| 129 – Pseudomonas alcaligenes | Pseudomonas alcaligenes NBRC 14159 | T |  | BATI0.1 |
| 130 – Pseudomonas denitrificans | Pseudomonas denitrificans ATCC13867 | T |  | CP004143.1 |
| 131 – Pseudomonas sp. | Pseudomonas nitroreducens TX1 | * |  | AMZB0.1 |
| 132 – Pseudomonas nitroreducens | Pseudomonas nitroreducens Aramco J | T |  | JUEH0.1 |
|  | Pseudomonas nitroreducens HBP1 | T |  | AZRU0.1 |
| 133 – Pseudomonas mendocina | Pseudomonas mendocina NBRC 14162 | T |  | BBQC0.1 |
|  | Pseudomonas mendocina S13 2 |  |  | JOVT0.1 |
|  | Pseudomonas mendocina S5 2 |  |  | AMCD0.2 |
| 134 – Pseudomonas sp. | Pseudomonas oleovorans MGY01 | * | <92%/P. oleovorans IFO 13583T | JNHE0.1 |
| 135 – Pseudomonas sp. | Pseudomonas mendocina DLHK | * |  | ALKM0.1 |
| 136 – Pseudomonas sp. | Pseudomonas pseudoalcaligenes AD6 | * | 94%/P. pseudoalcaligenes LMG 1225T rpoB  90%/P. pseudoalcaligenes IFO 1416T | JFJN0.1 |
| 137 – Pseudomonas sp. | Pseudomonas pseudoalcaligenes S1 | * |  | JTFL0.1 |
|  | Pseudomonas alcaliphila 34 | * | 94%/P. alcaliphila LMG 23134T gyrB | ANGB0.1 |
|  | Pseudomonas mendocina EGD−AQ5 | * |  | AVQF0.1 |
| 138 – Pseudomonas azotifigens | Pseudomonas azotifigens DSM 17556 | T |  | AUDU0.1 |
| 139 – Pseudomonas balearica | Pseudomonas stutzeri YC−YH1 | * |  | JUDR0.1 |
|  | Pseudomonas balearica DSM 6083 | T |  | GCA 000818015.1 |
| 140 – Pseudomonas sp. | Pseudomonas stutzeri TS44 | * |  | AJXE0.1 |
| 141 – Pseudomonas sp. | Pseudomonas stutzeri KF716 | * |  | BBQQ0.1 |
| 142 – Pseudomonas sp. | Pseudomonas stutzeri KOS6 | * |  | AMCZ0.2 |
| 143 – Pseudomonas sp. | Pseudomonas stutzeri CCUG 16156 | * |  | AGSL0.1 |
| 144 – Pseudomonas sp. | Pseudomonas stutzeri NF13 | * |  | AOBS0.1 |
| 145 – Pseudomonas chloritidismutans | Pseudomonas chloritidismutans AW−1 | T |  | AOFQ0.1 |
|  | Pseudomonas xanthomarina S11 | * |  | CCYE0.1 |
|  | Pseudomonas stutzeri ST−9 | * |  | JXJL0.1 |

**Table 1:** Genome clusters of *Pseudomonas* strains with suggested assignation. Mislabeled genomes are indicated with asterisk whereas type strains are labeled T. Correct assignation is left blank on assignation column.
